# Social determinants of mental health in Italy: the role of education in the comparison of migrant and Italian residents

**DOI:** 10.1186/s12939-022-01720-6

**Published:** 2022-08-23

**Authors:** Flavia Sesti, Valentina Minardi, Giovanni Baglio, Ruth Bell, Peter Goldblatt, Maurizio Marceca, Maria Masocco, Stefano Campostrini, Michael Marmot

**Affiliations:** 1 Italian Society of Migration Medicine, Rome, Italy; 2 Italian National Institute of Health, Rome, Italy; 3 Italian national Agency for Regional Health Services Rome, Rome, Italy; 4 UCL Institute of Health Equity, London, UK; 5 Sapienza University of Rome, Rome, Italy; 6 Ca’ Foscari University of Venice, Venice, Italy

**Keywords:** Depression, Immigrants, Surveillance, Mental health, Social determinants

## Abstract

Mental health is impacted by social, economic, and environmental factors, the Social Determinants of Health (SDH). Migrants experiencing precarious living and working conditions may be more at risk of poor mental health than the majority population.

This paper aims to evaluate the relationship of educational attainment and other SDH with depressive symptoms among the resident population, including Italians and migrants.

This study examined the respondents to the Italian “Progressi delle Aziende Sanitarie per la Salute in Italia” (PASSI) surveillance system, 2014–18. The sample of 144.055 respondents is composed of the resident working adults aged 25–69 with Italian citizenship (*n* = 136.514) and foreign citizenship (*n* = 7.491).

Findings show that among Italians high level of education appears to be a protective factor for mental health, in accordance with the international evidence (adjPR: tertiary education 0,74 *p*-value = 0.000). However, among immigrants high level of education is associated with the presence of depressive symptoms (adjPR: tertiary education: 1.61 *p*-value = 0.006), particularly for men (adjPR: tertiary education: 2.40 *p*-value = 0.006). The longer the length of stay in Italy for immigrants the higher the risk of depressive symptoms: adjPR for 10+ years: 2.23 *p*-value = 0.005.

The data show that high education could represent a risk factor for mental health of immigrants. Moreover, among migrants there are some significant mental health inequities between male and female related to the duration of stay in Italy, economic activity and educational level.

Considering that health is related to the nature of society as well as to access to technical solutions, multicultural societies require culturally oriented interventions for tackling health inequities. This means developing evidence-based policies in order to tackle health inequalities in the population as a whole, including culturally oriented measures in the larger framework of developing diversity sensitive services.

**Supplementary Information:**

The online version contains supplementary material available at 10.1186/s12939-022-01720-6.

## Introduction

It is well recognized that health inequalities are caused by the different conditions in which people are born, grow, live, work and age, driven by inequities in power, money and resources [1, 2]. These different personal, environmental and contextual conditions are usually referred to as Social Determinants of Health (SDH). While the effects of SDH on health outcomes are well recognized, less recent research examines the impact of SDH on migrant health, even though migrants are more likely than host populations to experience adverse conditions for health such as precarious living and working conditions [3].

While some countries including France and the United Kingdom have long experienced immigration, Italy has experienced immigration more recently.

During recent decades, Italy has registered an increasing number of immigrants: looking at data from the period of the study, Italy reached 5,255,503 residents immigrants at the end of 2018, which is 8.7% of the total population [4]. The IDOS Study and Research Centre estimated that about 1.3 million people born in Italy are second generation immigrants.

This migration has contributed to changes in the social structure [5], and presented a public health challenge [6].

However, it is recognized that integrating migrant health in health policies is an opportunity to improve public health and health systems [6, 7].

The global political agenda is focusing on migrant health; at the Seventy-second World Health Assembly in 2019 the Health Assembly discussed the Draft Global Action Plan ‘Promoting the health of refugees and migrants’ (2019–2023) [8], which followed the EUR/RC66/8 Strategy and action plan for refugee and migrant health in the WHO European Region [6]. Moreover, the 2030 Sustainable Development Goal Agenda recognizes the role of migrants for inclusive growth and sustainable development [9].

To improve migrant health, it is necessary to take SDH into account [3], considering that social and cultural issues are fundamental for addressing health equity in society as a whole [10].

Recognising this challenge, this paper aims to evaluate the interactions between some SDH and mental health among the resident population in Italy, comparing Italian born and immigrants.

## Methods

### Data source and study population

To evaluate how SDH could have different impacts on health outcomes, we used data from the Italian risk factor surveillance system “Progressi delle Aziende Sanitarie per la Salute in Italia” (PASSI), coordinated by the Italian National Institute of Health (ISS) and conducted by Regions and Local Health Units (LHUs). Since 2008, PASSI has been monitoring the prevalence of the major behavioural risk factors for chronic noncommunicable diseases and compliance level to the main preventive measures among the adult population (18–69 years of age) living in Italy. PASSI represents a useful tool for Regions and LHUs to describe the general population health profile, in order to plan health promotion and prevention interventions and monitor their effectiveness over time towards the objectives of the National Prevention Plan (NPP) [11].

In each LHU participating in the surveillance, the PASSI monthly sample is extracted by a random modality from an enrolment list of residents and is stratified by gender and age (18–34, 35–49, 50–69 years) in the same proportion as the reference population. Specially trained personnel from the LHUs’ public health departments administer telephone interviews using a standardised questionnaire. Eligibility PASSI criteria are: falling within the target age range (18–69 years), being reachable on a telephone number (landline or mobile), not being hospitalized nor institutionalized, understanding the Italian language (in the autonomous province of Bolzano the interviewees have the option of being interviewed in German) and having the ability to participate in the interview. The PASSI operational protocol encompasses the field-substitution technique among the same stratum of population. Once data are collected, they are anonymised and electronically recorded in a national database, and an annual dataset is created by aggregation of the interviews, which are finalised in the calendar year. Furthermore, multiple-year datasets are combined to ensure adequate sample size for allowing population subgroups explorations. PASSI methods for data collection have been described in detail elsewhere [12].

Since 2008 PASSI has provided surveillance of the health status of the resident adult population 18–69 years in all Italians regions, including in its sample frame migrants that are registered in the Health System Archives (registration is free and allows access to the universal services of the National Health System) [13].

Annual response rate has been always over 85%.

This study examined survey responses in the period January 2014 – December 2018, categorized by citizenship: Italians (95%) and foreigners (5%). Foreigners were then distinguished in two groups following the classification of the Italian National Institute for Statistics (ISTAT): immigrants from high emigration rate countries (PFPM, 4.8%) and immigrants from high-income countries (PSA, 0.2%).

The residing population under study is working adults aged 25–69 with Italian (*n* = 136.514) and PFPM (*n* = 7.491) citizenship for a total sample of 144.055 respondents. PFPM citizens come from Central and Eastern Europe, Africa, Asia with the exception of Israel and Japan, and Central -South America.

PFPM immigrants represent the majority of foreign population in Italy (95%), and they have been selected for this study considering that they represent the most vulnerable part of the migrant population in the PASSI sample.

### Outcome definition

On mental health, PASSI collects information only on depression symptoms, adopting the Patient Health Questionnaire 2 (PHQ-2), a 2-items depressive symptoms screening module [14], which is validated through the Structured Clinical Interview for Diagnostic and Statistical Manual of Mental Disorders (fourth edition) and the Italian version by D’Argenio et al. [15]. It shows a sensitivity of 87% and a specificity of 78% for major depressive disorders [16].

Specific items of the PASSI questionnaire were selected in order to investigate the association between SDH and this measure of mental health.

Information on sex, education (none/primary school, middle school, high school and, university), economic difficulties in making ends meet with the available financial resources (many, some or not at all), geographic residence area as categorised by the National Institute of Statistics criteria (North, Centre, South and major islands), living alone (yes/no), employment status (employed, searching for work and inactive) and, only for immigrants, length of stay in Italy (0–4 years, 5–9 years, 10 years and over) was examined in association with the presence or absence of depressive symptoms.

### Analysis

Descriptive data were presented as percentages. Complex survey design analyses using the Taylor series method for variance estimation were conducted. Prevalence of depressive symptoms estimates were obtained using a weighting system, assigning each record a probability weight equal to the inverse of the sampling fraction in each LHU stratum [12] . Adjusted Prevalence ratio (AdjPR) of symptoms of depression by citizenship (Italian or PFPM) and by demographic and socioeconomic characteristics were retrieved with multivariate Poisson regression model. All confidence intervals (CI) were calculated taking 95% as the confidence level and an alpha level of 0.05 was considered as significant. Statistical analyses were supported by Stata 15.0 (STATA Corp).

## Results

Data refer to the time period between 2014 and 2018, when 144,005 ITA and PFPM resident adult population aged 25–69 successfully responded to the SDH and mental health items, 49.6% of which were male (39.4% PFPM) and 50.4% female (60.6% PFPM) in 92 out of the 101 Italian LHUs (reference 2018) (Table 1). The response rate, calculated according to the guidelines of the American Association for Public Opinion Research (AAPOR 2001) and adjusted for ineligible cases, was above 81%.

**Table 1:** Distribution of sociodemographic characteristics of the sample, prevalence of depression and corresponding 95% confidence interval (CI), and Adjusted Prevalence ratio (AdjPR) and CI95% of symptoms of depression by citizenship (Italian or PFPM), by demographic and socioeconomic characteristics. PASSI 2014–2018 (*n* = 144,005)

	Italian population (25-69yy) (***n*** = 136,514)				PFPM population (25-69yy) (***n*** = 7491)			
Characteristics	Distribution (95% CI) (%)	Depression (95% CI) (%)	Adj PR^**a**^ (95%CI)	***p***-value	Distribution (95% CI) (%)	Depression (95% CI) (%)	Adj PR^**a**^ (95%CI)	***p***-value
**Overall**	–	6.3 (6.1–6.4)	–	–	–	4.9 (4.4–5.5)		
**Age group**								
25–34	18.0 (17.9–18.1)	4.4 (4.1–4.8)			28.8 (27.7–30.0)	4.6 (3.7–5.8)		
35–49	37.2 (37.1–37.3)	5.4 (5.1–5.7)			48.3 (47.0–49.6)	4.3 (3.6–5.0)		
50–69	44.8 (44.7–44.9)	7.7 (7.5–8.0)			22.9 (21.9–24.0)	6.6 (5.4–8.1)		
**Age, continuous**	–	–	1.011	0.000	–	–	1.009	0.125
**Gender**								
Male	49.6 (49.5–49.8)	4.7 (4.5–4.9)	1.000	–	39.4 (38.2–40.6)	3.7 (3.0–4.5)	1.000	
Female	50.4 (50.2–50.5)	7.8 (7.6–8.1)	1.493	0.000	60.6 (59.4–61.9)	5.7 (5.0–6.6)	1.570	0.002
**Educational attainment**								
Up to primary school	7.0 (6.8–7.2)	13.3 (12.2–14.5)	1.000	–	9.6 (8.8–10.5)	4.7 (3.3–6.7)	1.000	–
Lower secondary school	29.3 (29.0–29.7)	8.0 (7.6–8.4)	0.832	0.000	35.6 (34.3–36.9)	3.9 (3.1–4.8)	0.975	0.909
Higher secondary school	45.0 (44.6–45.3)	5.0 (4.8–5.2)	0.708	0.000	42.2 (40.9–43.5)	5.7 (4.9–6.8)	1.505	0.064
Tertiary education	18.7 (18.5–19.0)	4.3 (4.0–4.6)	0.739	0.000	12.6 (11.7–13.5)	5.6 (4.2–7.5)	1.614	0.064
**Economic difficulties**								
None	46.0 (45.7–46.4)	3.6 (3.4–3.8)	1.000	–	28.9 (27.7–30.1)	3.2 (2.4–4.2)	1.000	–
Some	40.1 (39.8–40.5)	6.2 (5.9–6.5)	1.678	0.000	47.7 (46.4–49.0)	4.3 (3.6–5.1)	1.427	0.039
Many	13.9 (13.6–14.1)	15.3 (14.6–16.1)	3.784	0.000	23.5 (22.3–24.7)	8.3 (7.0–9.8)	3.000	0.000
**Living alone**								
No	90.1 (89.9–90.3)	6.1 (5.8–6.2)	1.000	–	90.6 (89.8–91.4)	4.7 (4.2–5.3)	1.000	–
Yes	10.0 (9.8–10.2)	8.5 (7.9–9.2)	1.336	0.000	9.4 (8.6–10.2)	6.9 (5.1–9.4)	1.382	0.082
**Employment**								
Employed	63.7 (63.4–64.0)	4.5 (4.3–4.7)	1.000	–	66.9 (65.6–68.1)	3.9 (3.4–4.6)	1.000	–
Searching for work	8.1 (7.9–8.3)	9.7 (9.0–10.5)	1.440	0.000	14.9 (14.0–15.9)	8.3 (6.6–10.4)	1.598	0.002
Inactive	28.2 (28.0–28.5)	9.3 (8.9–9.7)	1.346	0.000	18.2 (17.2–19.3)	5.8 (4.6–7.2)	1.324	0.080
**Geographic area of residence**								
North	37.6 (37.5–37.7)	6.4 (6.1–6.6)	1.000	–	57.5 (56.3–58.8)	5.4 (4.7–6.1)	1.000	–
Centre	22.7 (22.6–22.8)	5.5 (5.2–5.7)	0.768	0.000	32.6 (31.4–33.8)	4.6 (3.7–5.7)	0.779	0.062
South and Islands	39.7 (39.6–39.8)	6.7 (6.3–7.0)	0.706	0.000	9.9 (9.0–10.9)	3.5 (2.2–5.3)	0.426	0.000
**Length of stay in Italy**								
0–4 years	–	–	–	–	7.6 (7.0–8.3)	2.8 (1.7–4.6)	1.000	–
5–9 years	–	–	–	–	22.3 (21.2–23.3)	4.1 (3.1–5.3)	1.645	0.106
10 years and over	–	–	–	–	70.1 (69.0–71.3)	5.5 (4.8–6.2)	2.233	0.005

^a^AdjPR Prevalence Ratio (Poisson) adjusted for socio-demographic variables (age in years, sex, geographical area of residence, educational attainment, economic difficulties, living alone, employment and length of stay for immigrants)

Table 2 shows the prevalence ratio (AdjPR) of symptoms of depression by sex and citizenship (Italian or PFPM), according to selected demographic and socioeconomic characteristics between 2014 and 2018. From our first analyses, the following major results emerge:

- Looking at SDH, tertiary level education is associated with a lower presence of depressive symptoms among Italians (AdjPR: 0.739) (Table 1), stratifying for gender this association is statistically significant only for women (AdjPR: 0.649), in accordance with the international evidence [17] (Table 2). Among migrants, tertiary level of education is associated with a higher presence of depressive symptoms (AdjPR for overall PFPM: 1.614, *p*-value = 0.064) (Table 1), especially for males (AdjPR: 2.401, *p*-value = 0.062) (Table 2). Among female migrants the AdjPR is 1.199 but it is not statistically significant (Table 2).
- Having economic difficulties is a confirmed risk factor for both Italians and PFPM (AdjPR many difficulties for Italians: 3.784 and for PFPM: 3.000, AdjPR some difficulties for Italians: 1.678 and for PFPM: 1.427) (Table 1).
- Employment status plays a relevant role, ‘looking for job’ is associated with a high risk of depressive symptoms in both Italians and PFPM (respectively AdjPR: 1.440 and 1.598) (Table 1). Inactive employment status among Italians is associated with a lower probability of depressive symptoms compared to immigrants (respectively AdjPR: 1.346 *p*-value =0.000 and 1.324 *p*-value = 0.080). In fact, PFPM inactive women are less likely to have depressive symptoms than PFPM men and Italian women (AdjPR for depressive symptoms among PFPM inactive women 1.000; for PFPM men: 3.260, and for Italian women: 1.082 (Table 2).
- Overall age it is not a risk factor for depressive symptom among males, but it is for Italian women (AdjPR: 1.015 per year) in accordance with the literature [18]. In contrast, it is not confirmed for migrant women (AdjPR: 1.004, *p*-value = 0.515) (Table 2).
- Living alone is also confirmed as a risk factor for mental health (AdjPR for Italians: 1.336 and for male PFPM: 2.619) (Table 1). In contrast, living alone was not a risk factor for depressive symptoms among PFPM women (AdjPR: 0.923) (Table 2).
- Area of residence: the data show a lower risk of depressive symptoms among Italians and migrants living in the centre and south Italy than the north (among Italians, AdjPR: 0.768 and 0.706 respectively) (Table 1). For migrants resident in Southern regions the risk of depressive symptoms is even lower (AdjPR: 0.426) (Table 1), especially among migrant women (AdjPR: 0.361) (Table 2).
- Among migrant women, depression seems also related to the length of stay in Italy (AdjPR for 5–9 years of stay: 2.047 and for 10+ years of stay: 2.768) (Table 2).

**Table 2:** Adjusted Prevalence ratio (AdjPR) and corresponding 95% confidence interval (CI) of symptoms of depression by sex and citizenship (Italian or PFPM), according to selected demographic and socioeconomic characteristics. PASSI 2014–2018 (*n* = 144,005)

	Italian male population (***n*** = 67,119)		Italian female population (***n*** = 69,395)		PFPM male population (***n*** = 2961)		PFPM female population (***n*** = 4530)	
Characteristics	Adj PR (95%CI)	***p***-value	Adj PR (95%CI)	***p***-value	Adj PR (95%CI)	***p***-value	Adj PR (95%CI)	***p***-value
**Age (yy)**	1.000 (0.994–1.005)	0.923	1.015 (1.011–1.019)	0.000	1.013 (0.992–1.035)	0.217	1.004 (0.991–1.018)	0.515
**Educational attainment**								
Up to primary school	1.000	–	1.000	–	1.000	–	1.000	–
Lower secondary school	1.023 (0.868–1.205)	0.789	0.762 (0.670–0.867)	0.000	1.480 (0.717–3.052)	0.289	0.822 (0.479–1.410)	0.476
Higher secondary school	0.879 (0.737–1.048)	0.150	0.636 (0.555–0.729)	0.000	2.948 (1.490–5.830)	0.002	1.122 (0.662–1.904)	0.668
Tertiary education	0.933 (0.749–1.162)	0.536	0.649 (0.548–0.769)	0.000	2.401 (0.959–6.011)	0.062	1.199 (0.658–2.185)	0.554
**Perceived economic difficulties**								
None	1.000	–	1.000	–	1.000	–	1.000	–
Some	1.769 (1.556–2.011)	0.000	1.629 (1.488–1.783)	0.000	1988 (0.878–4.501)	0.099	1.374 (0.946–1.997)	0.095
Many	5.036 (4.390–5.777)	0.000	3.196 (2.874–3.554)	0.000	6.332 (2.8884–13.903)	0.000	2.256 (1.486–3.425)	0.000
**Living alone**								
No	1.000	–	1.000	–	1.000	–	1.000	–
Yes	1.501 (1.322–1.704)	0.000	1.209 (1.084–1.350)	0.001	2.619 (1.504–4.560)	0.001	0.923 (0.578–1.475)	0.738
**Employment**								
Employed	1.000	–	1.000	–	1.000	–	1.000	–
Searching for work	1.577 (1.368–1.818)	0.000	1.239 (1.090–1.409)	0.001	2.472 (1.500–4.079)	0.000	1.171 (0.791–1.734)	0.431
Inactive	2.114 (1.835–2.435)	0.000	1.082 (0.989–1.184)	0.085	3.260 (1.478–7.191)	0.003	1.000 (0.727–1.376)	0.999
**Geographic area of residence**								
North	1.000	–	1.000	–	1.000	–	1.000	–
Centre	0.774 (0.688–0.870)	0.000	0.770 (0.709–0.837)	0.000	0.963 (0.599–1.547)	0.876	0.723 (0.531–0.985)	0.040
South and Islands	0.722 (0.646–0.808)	0.000	0.714 (0.654–0.779)	0.000	0.690 (0.324–1.470)	0.336	0.361 (0.206–0.632)	0.000
**Length of stay in Italy**								
0–4 years	–	–	–	–	1.000	–	1.000	–
5–9 years	–	–	–	–	0.952 (0.327–2.770)	0.928	2.047 (0.994–4.217)	0.052
10 years and over	–	–	–	–	1.442 (0.556–3.738)	0.451	2.768 (1.411–5.429)	0.003

Because educational systems can be very different among countries, the main nationalities of the countries of origin are introduced as a potential confounder into the model for immigrant’s sample analysis, for both men and women (Table S1). Accordingly to our previous results, high educational level remains associated with higher risk of depressive symptoms in PFPM population. However, immigrants coming from Asia seem to have a statistically significant reduction of risk to be depressed (AdjPR: 0.4) in comparison to those coming from the others high migratory pressure countries after adjustment for socio-demographic covariates; this protection is higher for women (AdjPR: 0.2).

## Discussion

Looking at the results, higher educational attainment could represent a risk factor for mental health of immigrants. In contrast to the Italian population who obtain a greater protective effect from higher education, a slight risk of depression was found among migrants with higher educational attainment (AdjPR for overall PFPM: 1.614, *p*-value = 0.064). Generally, international evidence shows the association between low educational levels and high risk of anxiety and depression. We speculate that this could be related to the failure of the migration project for some of the more educated migrants, for whom the migration experience failed to live up to their expectations and resulted in disappointment.

It may be that migrants with higher educational attainment are unable to access employment opportunities that match their skills or expectations. Employment conditions appear to play an important role in migrant health. Among both Italian men and migrant men those searching for work, or inactive in the labour market are more likely to report depressive symptoms than those in employment. For Italian women and migrant women this association was not present. Among migrants this issue could be related to a) culture of origin which may not fully recognize mental health problems and b) female migrants can be granted legal permission to stay in Italy on the grounds of family reunion, therefore they may have different expectations compared to male migrants [19].

Being a migrant and having a different cultural background are specific determinants of health, having a particular causation pathway influencing with socioeconomic position [20].

In PASSI surveillance system, we looked at more integrated migrants who contribute to the development of the country.

The length of stay in Italy may reflect the “exhausted migrant effect” [21] in mental health: the longer the period of time since migration, the higher the likelihood of developing depressive symptoms. As shown by Salami et al. in a study of migration and mental health in Canada, the better self-perceived mental health of migrants compared to Canadians experienced within the first 5 years of migration disappears with longer time since migration [22]. In the working sector for example, evidence show that migrants have a higher risk of work-related injuries over time compared to Italians [23]. This could also be due to the “assimilation process” [24] that sees migrants converging towards the relatively worse health condition of the host population. Still, it is relevant to notice how this convergence appears much higher among more educated migrants.

The analysis presents some limitations. It is important to outline that our study is based on self-reported information. Specifically, people may be less likely to report symptoms of depression because of the stigma surrounding mental illness, leading to an underestimation of their prevalence. For this reason, a validated population screening tool (PHQ2) was used instead of a self-reported diagnosis of depression that may be more affected by this type of phenomenon.

Moreover, considering the immigrants sample, all of them reside in Italy for a long period and they must have a sufficient knowledge of Italian to answer the telephone survey. In fact, the PASSI sample includes only about 70–75% of the migrants resident in Italy, which accounted for 8.7% of the total population in 2018. With regard to the country of origin, Europe is over-represented and Asia under-represented. To limit selection bias among more difficult-to-reach populations such as foreign residents, the PASSI protocol requires at least 6 contact attempts on different days of the week, including holidays, and in different time slots, including evenings.

In addition, younger people (under 18), elderly (over 69) and irregular migrants are not eligible for inclusion in the target population.

## Conclusion

The study showed that the risk of depressive symptoms varies between Italians and migrants according to social determinants, such as employment status and level of education. In particular, higher educational attainment appears to be a risk factor for depressive symptoms among migrants, but not among Italians.

Migrant mental health may be linked to the typically uncertain conditions of migrants related to aspects of daily life such as work permits and residency [25]. These issues characterize the integration process in the host country, and impact migrants’ wellbeing. Social and health workers can represent valuable supports facilitating the overcoming of cultural and social barriers, including critical aspects of the underqualified job.

Population health is related to the nature of society as well as access to health care [1, 3] Italy has experienced relatively recent cultural and societal changes, including migration. In recognition of the international focus on health equity within countries, there is a need to develop evidence-based policies to tackle health inequalities in the population as a whole. This means that multicultural societies need culturally oriented measures in the larger framework of diversity sensitive services. Long-term policies and structural adaptations of the health system are required for tackling health inequities among migrants [26].

In the insecurities of Bauman’s liquid modernity [27], within which society changes continuously, migration is a key element requiring measures aimed at promoting the art of living together [28], including the public health sector.

Considering that the study shows different patterns of risk for depressive symptoms, appropriate policy measures should be developed towards specific target populations such as the most educated migrants to prevent depression symptoms, to this aim social and health workers should work together in developing targeted models of care.

## Supplementary Information

**Additional file 1: Table S1.** Adjusted Prevalence ratio (AdjPR) and corresponding 95% confidence interval (CI) of symptoms of depression by sex, according to selected demographic and socioeconomic characteristics and macro areas of countries of origin for PFPM sample. PASSI 2014-2018 (*n*=144,005).

## Data Availability

The data presented in this study are available on request from the corresponding author. The data are not publicly available due to privacy restrictions.
