# Correction: Social determinants of mental health in Italy: the role of education in the comparison of migrant and Italian residents

**DOI:** 10.1186/s12939-022-01740-2

**Published:** 2022-09-30

**Authors:** Flavia Sesti, Valentina Minardi, Giovanni Baglio, Ruth Bell, Peter Goldblatt, Maurizio Marceca, Maria Masocco, Stefano Campostrini, Michael Marmot

**Affiliations:** 1 Italian Society of Migration Medicine, Rome, Italy; 2 Italian National Institute of Health, Rome, Italy; 3 Italian national Agency for Regional Health Services Rome, Rome, Italy; 4 UCL Institute of Health Equity, London, UK; 5 Sapienza University of Rome, Rome, Italy; 6 Ca’ Foscari University of Venice, Venice, Italy

**Correction: Int J Equity Health 21, 116 (2022)**

**https://doi.org/10.1186/s12939-022-01720-6**

Following the publication of the original article [1], we were notified that Professor Marceca was mistakenly called Massimo instead of Maurizio in the Authors' contribution section.

The original article has been corrected.
