# Supplementary material for: Social determinants of mental health in Italy: the role of education in the comparison of migrant and Italian residents
Source: Int J Equity Health. 2022 Aug 23;21:116. doi: 10.1186/s12939-022-01720-6 (PMC9400202; doi:10.1186/s12939-022-01720-6)
Supplement: Supplementary file 1 — Additional file 1: Table S1. Adjusted Prevalence ratio (AdjPR) and corresponding 95% confidence interval (CI) of symptoms of depression by sex, according to selected demographic and socioeconomic characteristics and macro areas of countries of origin for PFPM sample. PASSI 2014-2018 (n=144,005). [file 12939_2022_1720_MOESM1_ESM.doc]

**Table S1. Adjusted Prevalence ratio (AdjPR) and corresponding 95% confidence interval (CI) of symptoms of depression by sex, according to selected demographic and socioeconomic characteristics and macro areas of countries of origin for PFPM sample**. PASSI 2014-2018 (n=144,005).

|  | **PFPM population (n=7,490)** | | | | **PFPM male population**  **(n=2,961)** | | | **PFPM female population (n=4,529)** | |
| --- | --- | --- | --- | --- | --- | --- | --- | --- | --- |
| **Characteristics** | | **Adj PR**  **(95%CI)** | **p-value** | **Adj PR**  **(95%CI)** | | **p-value** | **Adj PR**  **(95%CI)** | | **p-value** |
| **Age (yy)** | | 1.009 | 0.142 | 1.014 | | 0.190 | 1.004 | | 0.538 |
| **Gender** | | | | | | | | | |
| Male | | 1.000 | - | 1.000 | | - | 1.000 | | - |
| Female | | 1.531  (1.161-2.018) | 0.003 | - | | - | - | | - |
| **Educational attainment** | | | | | | | | | |
| Up to primary school | 1.000 | | - | 1.000 | | - | 1.000 | | - |
| Lower secondary school | 0.967  (0.622-1.503) | | 0.880 | 1.436  (0.682-3.022) | | 0.340 | 0.804  (0.474-1.365) | | 0.420 |
| Higher secondary school | 1.473  (0.946-2.293) | | 0.086 | 3.041  (1.504-6.150) | | 0.002 | 1.050  (0.616-1.792) | | 0.857 |
| Tertiary education | 1.636  (0.986-2.714) | | 0.057 | 2.330  (0.927-5.859) | | 0.072 | 1.198  (0.660-2.174) | | 0.552 |
| **Perceived economic difficulties** | | | | | | | | | |
| None | 1.000 | | - | 1.000 | | - | 1.000 | | - |
| Some | 1.401  (1.002-1.959) | | 0.049 | 1.871  (0.836-4.190) | | 0.128 | 1.374  (0.950-1.987) | | 0.092 |
| Many | 2.913  (2.035-4.171) | | 0.000 | 5.756  (2.613-12.678) | | 0.000 | 2.233  (1.466-3.403) | | 0.000 |
| **Living alone** | | | | | | | | | |
| No | 1.000 | | - | 1.000 | | - | 1.000 | | - |
| Yes | 1.350  (0.936-1.947) | | 0.108 | 2.626  (1.511-4.563) | | 0.001 | 0.895  (0.557-1.436) | | 0.645 |
| **Employment** | | | | | | | | | |
| Employed | 1.000 | | - | 1.000 | | - | 1.000 | | - |
| Searching for work | 1.554  (1.150-2.100) | | 0.004 | 2.412  (1.470-3.959) | | 0.001 | 1.145  (0.773-1.697) | | 0.499 |
| Inactive | 1.313  (0.962-1.794) | | 0.087 | 3.159  (1.420-7.025) | | 0.005 | 0.997  (0.715-1.389) | | 0.986 |
| **Geographic area of residence** | | | | | | | | | |
| North | 1.000 | | - | 1.000 | | - | 1.000 | | - |
| Centre | 0.792  (0.599-1.047) | | 0.101 | 1.020  (0.603-1.726) | | 0.940 | 0.719  (0.522-0.989) | | 0.043 |
| South and Islands | 0.436  (0.274-0.694) | | 0.000 | 0.710  (0.328-1.538) | | 0.386 | 0.367  (0.209-0.647) | | 0.001 |
| **Length of stay in Italy** | | | | | | | | | |
| 0–4 years | 1.000 | | - | 1.000 | | - | 1.000 | | - |
| 5–9 years | 1.586  (0.867-2.904) | | 0.135 | 0.931  (0.318-2.722) | | 0.896 | 1.928  (0.934-3.981) | | 0.076 |
| 10 years and over | 2.148  (1.225-3.765) | | 0.008 | 1.369  (0.525-3.572) | | 0.521 | 2.621  (1.332-5.155) | | 0.005 |
| **Macro areas of countries of origin** | | | | | | | | | |
| EU countries* | 1.000 | | - | 1.000 | | - | 1.000 | | - |
| Other European countries (not EU)** | 0.938  (0.685-1.283) | | 0.687 | 1.695  (0.949-3.127) | | 0.091 | 0.763  (0.518-1.122) | | 0.169 |
| Northern Africa | 1.125  (0.750-1.690) | | 0.568 | 1.513  (0.690-3.324) | | 0.301 | 1.044  (0.639-1.707) | | 0.864 |
| Sub-Saharan Africa | 1.095  (0.652-1.840) | | 0.732 | 1.516  (0.653-3.520) | | 0.333 | 0.884  (0.438-1.784) | | 0.730 |
| Asia | 0.368  (0.195-0.694) | | 0.002 | 0.781  (0.299-2.040) | | 0.614 | 0.240  (0.095-0.609) | | 0.003 |
| America | 0.995  (0.668-1.481) | | 0.979 | 0.812  (0.280-2.352) | | 0.701 | 0.988  (0.645-1.514) | | 0.956 |

* Bulgaria, Malta, Poland, Romania, Hungary, Estonia, Latvia, Lithuania, Croatia, Slovenia, Slovakia, Czech Republic, Cyprus

** Albania, Ukraine, Russia, Bosnia and Herzegovina, Republic of Macedonia, Moldova, Belarus, Serbia, Montenegro, Kosovo, Turkey
